# *Mycobacterium tuberculosis* lacking CtpF and MmpL7 transporters exhibits altered microbiological traits associated with virulence determinants

**DOI:** 10.1007/s00203-026-04921-7

**Published:** 2026-05-09

**Authors:** Sergio Ricaurte-Ruiz, Alver Cruz-Cacais, Vanessa Vásquez-Godoy, Milena Maya-Hoyos, Carlos Y. Soto

**Affiliations:** Chemistry Department, Faculty of Sciences, Universidad Nacional de Colombia, Carrera 30 # 45-03, Ciudad Universitaria, Bogotá, Colombia

**Keywords:** *Mycobacterium tuberculosis*, P-type ATPases, CtpF, MmpL7, PDIM, Immunomodulatory antigens

## Abstract

Tuberculosis (TB) remains a major global public health problem, emphasizing the necessity of identifying attenuation targets in *Mycobacterium tuberculosis* (*Mtb*) for the development of novel control strategies. In this context, plasma membrane transporters are critical determinants of *Mtb* virulence, including CtpF, a Ca^2+^ P-type ATPase, and MmpL7, a translocator of phthiocerol dimycocerosate (PDIM). In this study, we characterized in vitro the microbiological features of the *Mtb*H37RvΔ*ctpF* and *Mtb*H37RvΔ*mmpL7* strains. Both mutants displayed reduced colony roughness and hydrophobicity compared to *Mtb*H37Rv, suggesting alterations in the composition of cell wall lipids. Chromatographic analyses confirmed changes in the lipid profiles; *Mtb*H37RvΔ*ctpF* exhibited altered phosphatidylinositol mannosides and trehalose dimycolates profiles, whereas *Mtb*H37RvΔ*mmpL7* showed accumulated PDIM intracellularly. Moreover, transcriptional analyses revealed the downregulation of PDIM biosynthesis and transport genes in both mutants. However, both strains retained the ability to secrete early secreted antigenic target-6 (ESAT-6) and culture filtrate protein-10 (CFP-10), indicating that their immunostimulatory potential was preserved. These findings provide in vitro evidence that deletions of *ctpF* and *mmpL7* alter envelope-associated traits commonly linked to mycobacterial virulence.

**Graphical abstract:**

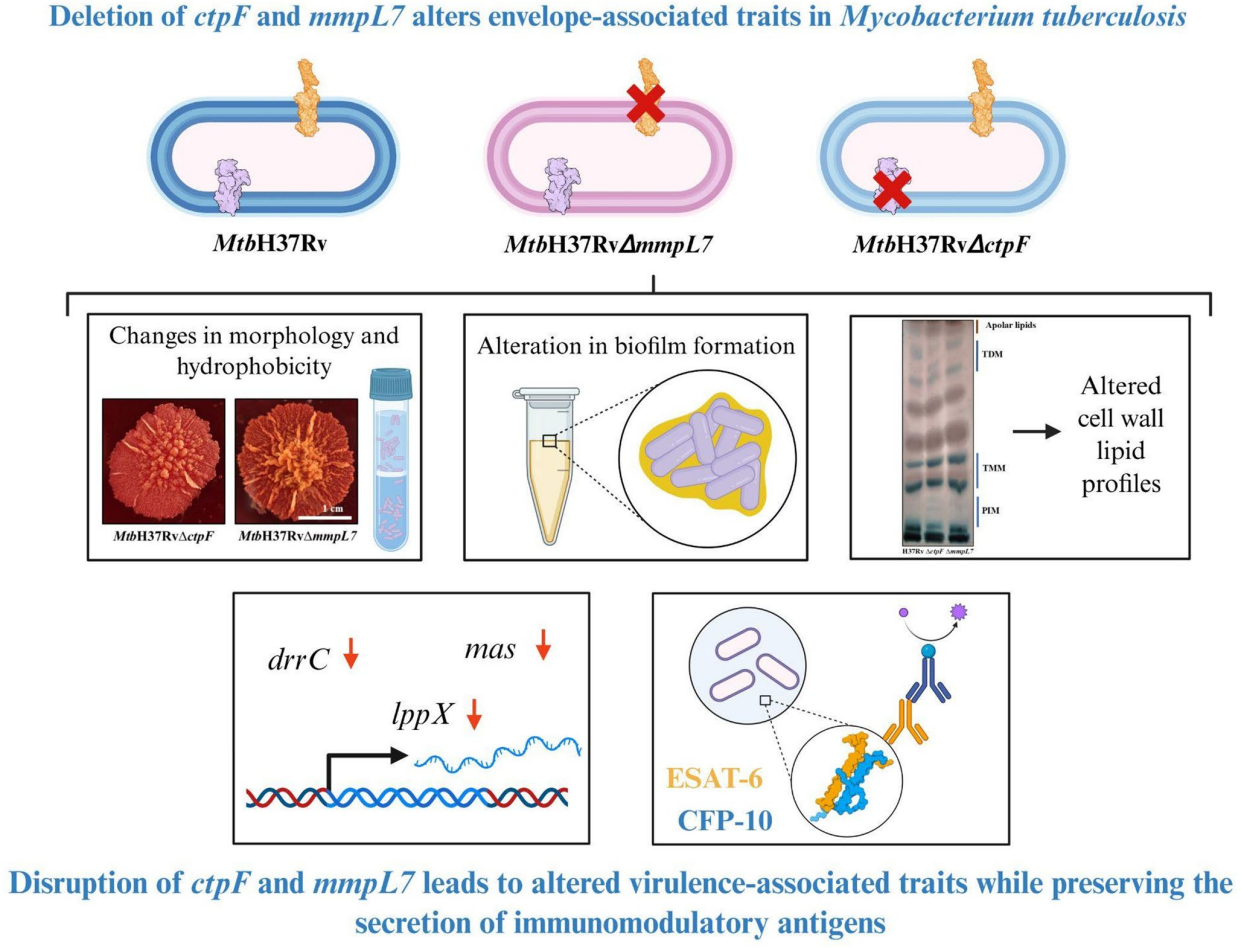

**Supplementary Information:**

The online version contains supplementary material available at 10.1007/s00203-026-04921-7.

## Introduction

Tuberculosis (TB) is still one of the world’s leading health challenges. According to the World Health Organization (WHO), more than 10 million new cases and approximately 1.3 million deaths were reported in 2024. This reflects a resurgence associated with the disruption of TB control programs during the COVID-19 pandemic. The global burden of TB is further exacerbated by the emergence and dissemination of multidrug-resistant and extremely drug-resistant (MDR and XDR) *Mtb *strains, as well as the frequent coinfection with human immunodeficiency virus (HIV), both of which compromise the effectiveness of current therapeutic regimens (World Health Organization 2025). This underscores the urgent need for preventive measures that are both effective and long-lasting.

Since its introduction in 1921, Bacillus Calmette-Guérin (BCG), an attenuated strain of *Mycobacterium bovis*, has remained the only licensed vaccine against TB (Pai et al. 2016). BCG provides consistent protection against disseminated TB in infants. However, its efficacy against pulmonary TB in adolescents and adults is highly variable (0–80%) (Pai et al. 2016; Lai et al. 2023). A key contributor to this variability is the progressive loss of genomic regions during the attenuation process of BCG (Gröschel et al. 2016). In addition, the efficacy of BCG is influenced by several factors, including the geographic setting, the host’s genetic background, and the variability among BCG strains (Lai et al. 2023). Notably, the absence of immunodominant antigens such as the early secretory antigenic target 6 (ESAT-6) and the culture filtrate protein 10 (CFP-10), both critical for eliciting robust T-cell-mediated immune responses (Brandt et al. 1996; Wu et al. 2008), has been linked to the reduced protective capacity of the vaccine (Maue et al. 2007).

The development of next-generation vaccines against TB has become a global priority. Live-attenuated *Mtb* strains represent a promising alternative, as they can preserve a broad antigenic repertoire (Martin et al. 2020; Romano et al. 2023), which elicits stronger and longer-lasting memory responses (Brandt et al. 1996; Wu et al. 2008). However, the rational design of a vaccine requires identifying molecular targets whose attenuation reduces virulence without compromising immunogenicity.

In this regard, transmembrane proteins constitute attractive targets for attenuation of *Mtb*. Among them, P-type ATPases are essential for metal ion homeostasis and for adaptation to phagosomal stress (Novoa-Aponte and Soto Ospina 2014; Maya-Hoyos et al. 2019; Garg et al. 2020). Transcriptional studies have shown that several of these enzymes are upregulated under hypoxic conditions, upon exposure to toxic compounds, and during infection (Novoa-Aponte and Soto Ospina 2014). A notable example is CtpF, which regulates Ca^2+^ homeostasis by preventing cytoplasmic overload and is markedly expressed during infection to promote *Mtb* survival (Novoa-Aponte and Soto Ospina 2014; Maya-Hoyos et al. 2019). Previous studies have shown that a mutation in the *ctpF* gene significantly reduces the bacterial burden in macrophages and prolongs host survival in a murine model. CtpF has also been shown to contribute to *Mtb* dormancy (Maya-Hoyos et al. 2022).

Another relevant family of transmembrane proteins is the MmpL, which mediates the export of lipids to the cell envelope and contributes to both impermeability and immune evasion (Rens et al. 2021). Specifically, MmpL7 transports phthiocerol dimycocerosate (PDIM), which is a well-established modulator of host–pathogen interactions. Importantly, PDIM does not act as an independent virulence determinant, but rather functions in coordination with the ESAT-6 secretion system 1 (ESX-1). This secretion system mediates the secretion of key effector proteins such as ESAT-6 and CFP-10, which are directly involved in phagosomal membrane disruption. In this context, PDIM has been shown to facilitate ESX-1–dependent membrane perturbation and cytosolic access, highlighting a coordinated mechanism underlying host–pathogen interaction (Barczak et al. 2017; Augenstreich et al. 2017). PDIM also influences inflammatory cell recruitment and granuloma dynamics during early infection (Cambier et al. 2014). Furthermore, PDIM contributes to *Mtb* virulence by masking pathogen-associated molecules from recognition by host innate immune receptors (Cambier et al. 2014). In addition, studies using an *mmpL7* mutant strain have demonstrated a significant reduction in autophagy in *Mtb*-infected cells and have shown that MmpL7 is required for virulence in mouse models (Domenech et al. 2005; Astarie-Dequeker et al. 2009; Melly and Purdy 2019).

Despite these findings, important questions remain regarding how the absence of CtpF and MmpL7 may affect the physiology of the mycobacterial envelope, as well as its interaction with the host and modulation of the immune response. Addressing these issues is critical for determining their suitability as attenuation targets.

Therefore, the present study was designed as an in vitro framework to examine microbiological traits associated with virulence, as a first step toward future validation in infection models. In this context, the selection of the *Mtb*H37RvΔ*ctpF* and *Mtb*H37RvΔ*mmpL7* mutants was based on their established roles in calcium homeostasis and PDIM transport, respectively. These are two functionally distinct processes that are critically involved in mycobacterial virulence and host–pathogen interaction.

## Materials and methods

### Bacterial strains and growth conditions

The bacterial strains used in this study are listed in Table S1 (Supplementary Material). All strains were maintained as glycerol stocks at − 80 °C. *Escherichia coli* (*E. coli*) was cultured with agitation in Luria-Bertani (LB) broth or on LB agar at 37 °C for 16 h. Strains harboring plasmids were supplemented with the appropriate antibiotic at the following concentrations: 100 µg/mL ampicillin (Amp), 100 µg/mL hygromycin (Hyg), and 25 µg/mL kanamycin (Kan). In experiments employing blue-white selection based on β-galactosidase activity, culture media were supplemented with 80 µg/mL 5-bromo-4-chloro-3-indolyl-β-D-galactopyranoside (X-Gal) and 0.5 mM isopropyl-β-D-thiogalactopyranoside (IPTG).

For the hydrophobicity assay, congo red staining, colony spreading, biofilm formation assay and RNA isolation, the mycobacterial strains (*Mtb*H37Rv, *Mtb*H37RvΔ*ctpF*, *Mtb*H37RvΔ*mmpL7*) were grown in Middlebrook 7H9 broth (catalog number 271310, Becton Dickinson, NJ, USA) supplemented with (10% v/v; 25 µg/mL oleic acid, 0.5% bovine albumin fraction V, 0.2% dextrose, 0.004% catalase) OADC, 0.5% glycerol (catalog G5516, Sigma-Aldrich, MO, USA), and 0.05% tyloxapol (Tyl) (catalog T8761, Sigma-Aldrich), at 37 °C with agitation (80 rpm) until Optical Density at 600 nm (OD_600_) ≈ 1.0. For biofilm quantification and ESAT-6/CFP-10 detection, cultures were grown in modified Sauton broth (0.5 g/L K_2_HPO_4_, 0.5 g/L MgSO_4_, 4 g/L L-asparagine, 0.05 g/L ferric ammonium citrate, 4.76% glycerol, 0.2% glucose, 1 mg/L ZnSO_4_; pH ≈ 7.0) incubated statically for 6 weeks (for biofilms) or with agitation (80 rpm) for 5 weeks (secreted and total proteins extraction). When required, 7H9, 7H10, and 7H11 were supplemented with antibiotics at the following final concentrations: 100 µg/mL Hyg, 25 µg/mL Kan, and 10 µg/mL cycloheximide (Chx).

### Construction of the *mmpL7*-defective *Mtb* strain

The *mmpL7* gene was deleted from *Mtb*H37Rv using the Che9c recombineering system as described by van Kessel and Hatfull (Van Kessel and Hatfull 2007). *Mtb* genomic DNA was isolated using the CTAB-NaCl/proteinase K method with modifications (Parish and Roberts 2015). In summary, the allelic exchange substrate (AES) was constructed by independently cloning 500 bp fragments corresponding to the upstream and downstream regions of the *mmpL7* gene into the pYUB854 vector, flanking a Hyg resistance cassette, to generate the pVVG4 plasmid. The *mmpL7* AES (3048 pb) was excised from pVVG4 through double digestion with BspHI and XhoI.

In parallel, the recombineering *Mtb* strain (*Mtb*H37Rv::pJV53) was grown in 7H9 medium supplemented with 0.2% succinate, 0.05% Tween 80, and Kan, until an OD_600_≈0.8. Then, 0.2 M glycine was added, and recombinase expression was induced by the addition of 0.2% acetamide. These cells were electroporated with 200 ng of AES and plated on 7H11-OADC-Kan-Hyg (Van Kessel and Hatfull 2007; Maya-Hoyos et al. 2019). From *Mtb* colonies, the deletion of the *mmpL7* gene was confirmed by PCR using the primers listed in Table S1 (Supplementary Material). The mutant strain was purified through successive passages. This process consisted of inoculating 1 µL of culture from the original colony into 10 mL of 7H9-OADC-Chx-Hyg. The culture was then incubated for 21 days, and this procedure was repeated three additional times. From the final culture, serial dilutions (10^− 1^ to 10^− 5^) were prepared, and 100 µL of each dilution was plated onto 7H10-OADC-Chx-Hyg until colonies appeared. The colonies obtained were evaluated by PCR to determine their purity (data not shown). Finally, complementation experiments were performed using pMV261, a replicative vector under the control of the constitutive *hsp60* promoter. However, wild-type (WT) phenotypes were not restored, likely due to overexpression effects. Therefore, complementation was not included in the present analysis.

## Congo red staining

3 µL of *Mtb* cells grown in 7H9-OADC-Tyl (until OD_600_≈ 1.0) were inoculated in the center of 7H10-OADC-Chx supplemented with 100 µg/mL Congo Red. The plates were incubated at 37 °C without agitation for 4 weeks. The colony morphology was visually classified as rough or smooth according to (Julián et al. 2010),and further categorized as opaque or glossy, and pigmented or non-pigmented based on the presence of Congo Red dye (Maya-Hoyos et al. 2015). Colony morphology was evaluated from three biological replicates, each comprising five technical replicates for each strain. Morphology was compared among strains using *Mtb*H37Rv as a reference, with particular attention to the relative presence of cord-like structures on the colony surface, a serpentine growth pattern characteristic of pathogenic strains (Darzins 1958; Hunter et al. 2006).

## Hydrophobicity assay

The hydrophobicity assay was performed as reported by Jankute et al. 2017; with modifications (Jankute et al. 2017). *Mtb* grown in 7H9-OADC-Tyl (until OD_600_≈1.0) were left at room temperature without agitation for one week until biomass sedimentation. After, the supernatant was removed and the biomass was heat-inactivated at 95 °C for 1 h. Cells were washed once with PUM buffer (2.2 % K_2_HPO_4_·3H_2_O, 0.726% KH_2_PO_4_, 0.18% urea, 0.02% MgSO_4_·7H_2_O, pH ≈ 7.1) and resuspended in 0.05% PUM-Tween 80 to an OD_600_≈  0.7 (A_0_). Then, an aliquot of 3 mL was transferred to a glass tube containing 2.4 mL of hexadecane, mixed by inversion, and incubated at 37 °C for 15 min. Phase separation was allowed at room temperature for 15 min. The upper phase was removed, and 1 mL of the aqueous phase was collected to measure OD_600_ (A_1_). Hydrophobicity was calculated as follows:

% Hydrophobicity= (1–(A_1_/A_0_))×100. The test was performed with two biological replicates and three technical replicates. Statistical analysis was performed using one-way analysis of variance (ANOVA) followed by Tukey’s test.

## Biofilm formation assay

100 µL of *Mtb* grown in 7H9-OADC-Tyl (until OD_600_≈1) were inoculated into microtubes containing modified Sauton medium (Ojha et al. 2008; Richards et al. 2019). Tubes were incubated at 37 °C without agitation for 6 weeks. Planktonic cells were removed by tilting the tubes at 90°. Tubes were washed once with 1X PBS (137 mM NaCl, 2.7 mM KCl, 10 mM Na_2_HPO_4_, 1.8 mM KH_2_PO_4_; pH ≈ 7.4) to remove residual cells. Then, 0.1% crystal violet was added and left at room temperature for 15 min. Tubes were centrifuged at 13,200 rpm for 10 min, and the supernatants were discarded. Biofilm-bound dye was extracted by adding absolute ethanol and incubating for 10 min at room temperature. For quantification, 100 µL of the ethanol-dye extract from each tube was mixed with 100 µL of absolute ethanol in a 96-well plate. Next, plates were incubated at room temperature for 24 h, and absorbance was measured at 570 nm in an iMARKTM Microplate Reader (Bio-Rad, CA, USA). Wells containing only absolute ethanol were used as a negative control. The experiment was performed using biological (*n* = 4) and technical (*n* = 8) replicates per *Mtb* strain. Statistical analysis was performed using one-way ANOVA followed by Tukey’s test.

## Colony spreading assay

Briefly, 3 µL of *Mtb* cultures in 7H9-OADC-Tyl (until OD_600_≈1) were inoculated at the center of a plate containing 7H9-OADC solidified with 0.4% agarose. Plates were incubated at 37 °C for 4 weeks (Maya-Hoyos et al. 2015). Colony spreading was monitored every 3 days by measuring the diameter of radial growth from the inoculation site. The assay was performed with five biological replicates. Data were analyzed using one-way ANOVA followed by Tukey’s test. 

### Lipid extraction and thin-layer chromatography (TLC) analysis.

Lipid extraction was performed for mycobacteria grown on 7H9-OADC solidified with 0.4% agarose. The colonies were scraped from the agarose surface and transferred to screw-cap glass tubes. Then, 5 mL of petroleum ether was added to the cells and incubated at room temperature for 3 min. After gentle mixing, the supernatant was collected and immediately mixed with 5 mL of chloroform: methanol and incubated for 24 h. Afterwards, total lipids were extracted from the remaining biomass (after ether extraction) by incubation with 10 mL of (1:2) chloroform: methanol, then (2:1), each for 24 h at 37 °C with agitation (80 rpm). Lipid extracts were filtered, dried at 65 °C, and resuspended in 20 mg/mL chloroform (Guallar-Garrido et al. 2021).

For chromatography analysis, 400 µg of lipid extract was spotted onto TLC plates (Merck Silica Gel G60 F254, 20 × 20 cm, glass-backed). For PDIM analysis, (9:1, v/v) petroleum ether/diethyl ether was used as the mobile phase. For glycolipid separation, a (65:25:4, v/v/v) chloroform/methanol/water eluent system was used (Lefebvre et al. 2018; Krishnamoorthy et al. 2021; Jones et al. 2024). TLC plates were visualized by spraying with 10% phosphomolybdic acid in ethanol for the detection of PDIM and 1% anthrone in concentrated sulfuric acid for the identification of glycolipids. Finally, TLC plates were heated at 140 °C for 15 min to visualize lipid spots (Camacho et al. 2001; Maya-Hoyos et al. 2015; Guallar-Garrido et al. 2021).

### RNA isolation and cDNA synthesis

*Mtb* strains grown on 7H9-OADC (until OD_600_≈1) were collected by centrifugation at 13,000 rpm for 10 min, washed with diethyl pyrocarbonate (DEPC)-treated H_2_O, and the cell pellets were used for RNA isolation using the TRIzol method (Invitrogen, Carlsbad, CA, USA) based on the Rustad method (Rustad et al. 2009). RNA was resuspended in 60 µL of DEPC-treated H_2_O, quantified in a NanoDrop™ OneC (Thermo Fisher Scientific, Waltham, MA, USA), and its integrity was evaluated on 2% agarose gels.

To eliminate DNA contamination, 2 µg of RNA was treated with 4 µL of DNase I (EN0521 Thermo Scientific) and 1 µL of RNase inhibitor (N8080119 Thermo Fisher Scientific, Waltham, MA, USA) at 37 °C for 30 min. Then, DNase was inactivated by adding 2 µL 25 mM EDTA and incubating at 65 °C for 10 min. cDNA synthesis was carried out using 2 µg of RNA-treated, 1 µL of 10 mM dNTPs, 1 µL of 0.2 µg/µL random primers (Thermo Fisher Scientific), 1 µL of 0.02 mM gene-specific reverse primers, and 1 µL of OneScript^®^ Plus Reverse Transcriptase (abm). The reaction was performed according to the manufacturer’s instructions. The cDNA was stored at − 20 °C until use.

## qRT-PCR analysis

The transcription levels of the genes *16SrRNA* (normalizer), *lppX*, *drrC*, and *mas* were quantified using the Pfaffl method (Pfaffl 2001; Pfaffl et al. 2004). Quantifications were performed for triplicate samples, including a negative control (without cDNA addition). The amplification efficiency of target and reference genes was determined using 10-fold serial dilutions of *Mtb*H37Rv genomic DNA. qPCR and qRT-PCR were performed using the SsoAdvanced Universal SYBR Green Supermix of Bio-Rad on the CFX-96 thermocycler (Bio-Rad Laboratories, Hercules, CA, USA). Cycling conditions were as follows: initial denaturation at 95 °C for 5 min, followed by 39 cycles of 95 °C for 15 s, melting temperature (T_m_) (°C) for 10 s, and 72 °C for 15 s.

## ESAT-6 and CFP-10 protein production

The presence of ESAT-6 and CFP-10 was evaluated in whole-cell extracts and culture supernatants. For this, 100 mL of mycobacterial culture was centrifuged at 3000 rpm for 15 min, and the pellet was separated from the culture supernatant. First, the supernatants were filtered through a 0.22-µm filter, and the proteins were precipitated by adding four volumes of cold acetone at − 20 °C overnight. The bacterial pellets were resuspended in 1 mL 1X PBS and washed twice. The PBS was removed by centrifugation, and the cellular pellets were heat-inactivated (95 °C) for 60 min, and lysed either by bead beating or sonication.

For bead beating, pellets were resuspended in lysis buffer (50 mM NH_4_HCO_3_, 10 mM MgCl_2_, 0.1% NaN_3_, 1 mM EDTA, 7 M urea, 2 M thiourea, 5 mM DTT, pH ≈ 7.4; 1 mL of lysis buffer per 25 mg of pellet) and disrupted with zirconia beads (six pulses of 30 Hz, with cooling between pulses). For sonication, the pellets were resuspended in lysis buffer (1 mL of lysis buffer per 25 mg of pellet) and sonicated for 15 min on ice (80% duty, output 3). Then, they were kept at 4 °C for 5 min, and then sonicated again under the same conditions. In both lysates, proteins were precipitated with 25% trichloroacetic acid at 4 °C overnight. Then, the lysates were centrifuged (15 min, 13000 rpm, 4 °C), and the proteins were washed twice with 250 µL of cold water and 1 mL of cold acetone (Rabodoarivelo et al. 2016). Finally, the protein extracts were resuspended with 50 mM NH_4_HCO_3_ and 1 M urea (200 µL per 25 mL of filtrate or 250 µL per whole-cell extract).

The protein concentration was determined using the Bradford method (OD_595_/OD_450_) (Zor and Selinger 1996). Before SDS-PAGE, the samples were treated with 10 M urea and 6X loading buffer (35% Tris/SDS, 30% Glycerol, 10% SDS, 33% 2-mercaptoethanol (reducing conditions), 0.012% bromophenol blue, pH ≈ 6.8) and then heated to 90 °C for 5 min.

Western blot analysis was carried out using standard procedures. Primary antibodies (Anti-*Mycobacterium tuberculosis* ESAT-6 Antibody (A86648), antibodies.com) or (Anti-*Mycobacterium tuberculosis* CFP-10 Antibody (A86662), antibodies.com) were used at a concentration of 0.5 µg/mL. The secondary antibody (Anti-Rabbit IgG (whole molecule)–Alkaline Phosphatase antibody, produced in goat; Sigma-Aldrich) was used at a 1:20000 dilution. The membranes were revealed using 0.165 mg/mL 5-bromo-4-chloro-3-indolyl-phosphate (BCIP) and 0.33 mg/mL nitro blue tetrazolium (NBT) in buffer (100 mM Tris-HCl, 150 mM NaCl, 1 mM MgCl_2_; pH ≈ 9.0).

## Results

### Deletion of the *mmpL7* gene in the *Mtb*H37Rv strain

**Fig. 1:**
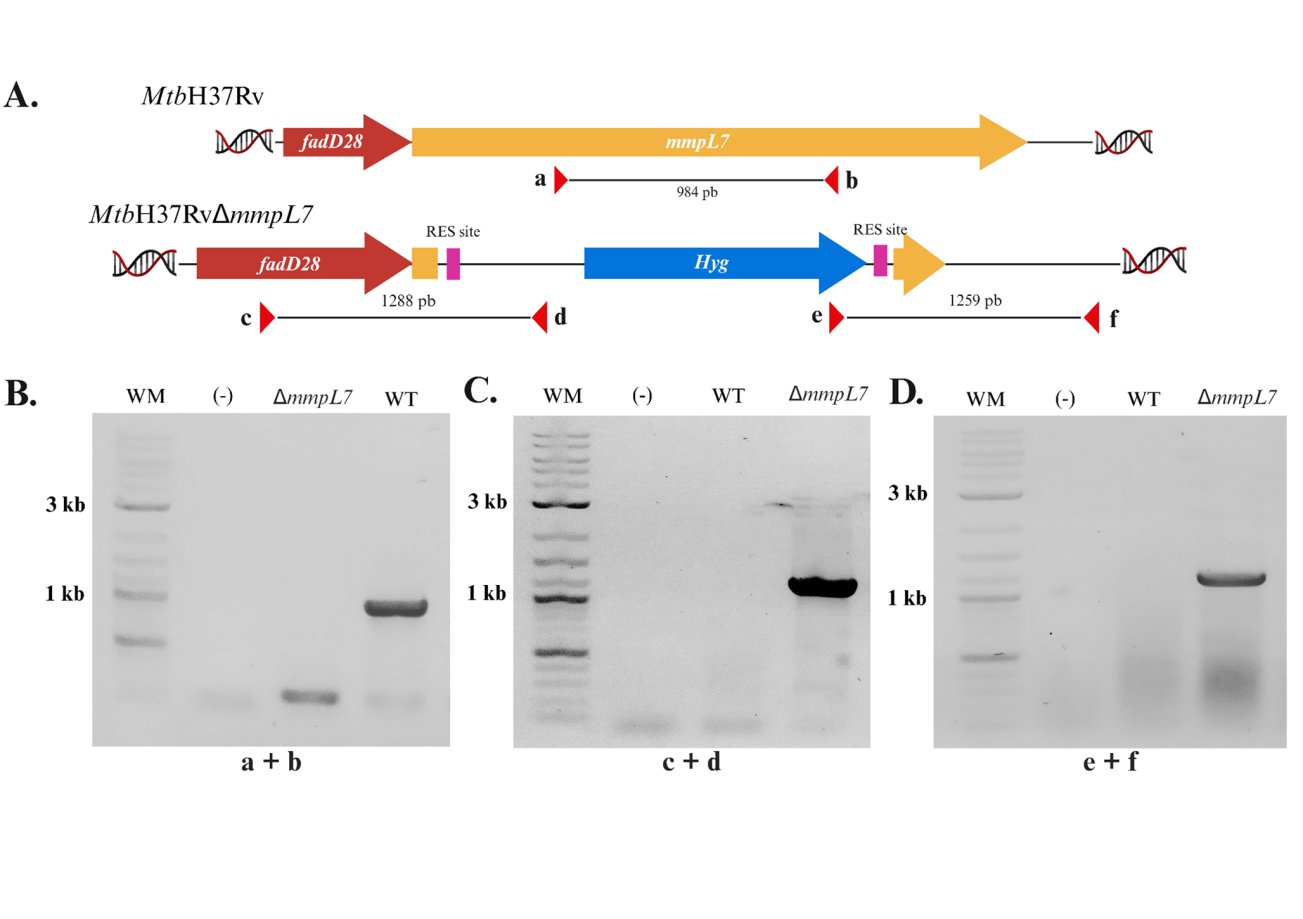
Verification of the *mmpL7* gene mutation in *Mtb*H37Rv. **A.** Schematic representation of the genomic region in the WT and *Mtb*H37RvΔ*mmpL7* strains, showing the location of the primers (red tips) used for PCR (Diagram created in https://BioRender.com). **B.** PCR verification of the *mmpL7* gene deletion using primers a and b (984 bp amplicon) (**C–D**). PCR confirmation of the mutant genotype and *Hyg* resistance cassette inserted at the target locus in *Mtb*H37RvΔ*mmpL7*. Primers c and d yield a 1288 bp product, while primers e and f generate a 1259 bp fragment. WM: molecular weight marker (GeneRuler™ 1 kb DNA Ladder, Thermo Scientific). (–): no DNA template

The *Mtb*H37RvΔ*mmpL7* strain was constructed to evaluate the impact of *mmpL7* disruption on key microbiological virulence-associated traits of *Mtb*. The absence of *mmpL7* was verified by PCR using primers designed within the gene sequence (primers a and b). As expected, a 984 bp amplicon was obtained from the WT strain (*Mtb*H37Rv), whereas no amplification was detected in the mutant, confirming successful deletion of *mmpL7* (Fig. 1A and B). The presence of the AES in the *Mtb*H37RvΔ*mmpL7* strain was further confirmed by PCR using primers that annealed within the AES and to genomic regions flanking the target locus (Fig. 1C and D). As expected, amplicons of 1288 bp (primers c and d) and 1259 bp (primers e and f) were obtained exclusively from the mutant strain, while no products were amplified from the *Mtb*H37Rv.

### Mutations of the *ctpF* and *mmpL7* genes alter colony morphology and hydrophobicity of *Mtb*H37Rv cells

**Fig. 2:**
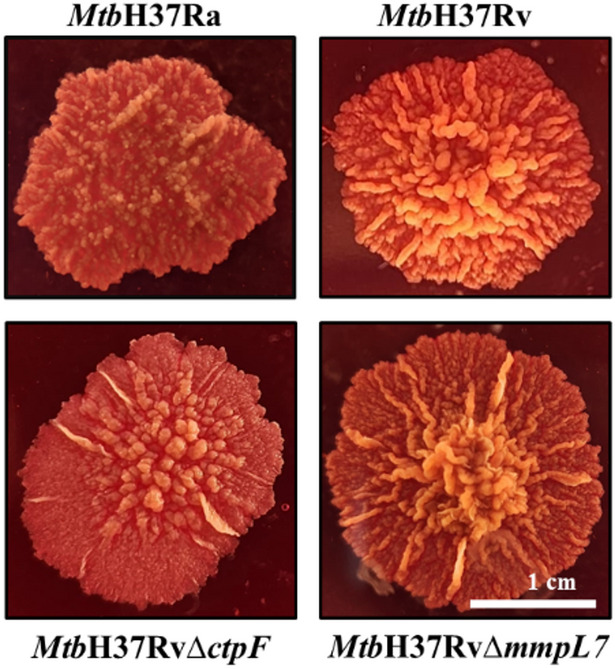
Colony morphology of *Mtb*H37Ra, *Mtb*H37Rv, *Mtb*H37Rv∆*ctpF*, and *Mtb*H37Rv∆*mmpL7*. *Mtb* strains were grown on 7H10-OADC-Congo Red. A white line represents one centimeter on the scale

Colonies of the *Mtb*H37RvΔ*ctpF*, *Mtb*H37RvΔ*mmpL7*, and *Mtb*H37Rv strains exhibited a dry, opaque, and irregular morphology with curled edges, firm texture, and elevated structure (Fig. 2). However, differences in colony roughness and morphology were observed among the strains. Specifically, mutant strains exhibited lower levels of the serpentine cord factor than *Mtb*H37Rv, which is the reference strain for a virulent phenotype (Hunter et al. 2006; Lerner et al. 2020).

**Fig. 3:**
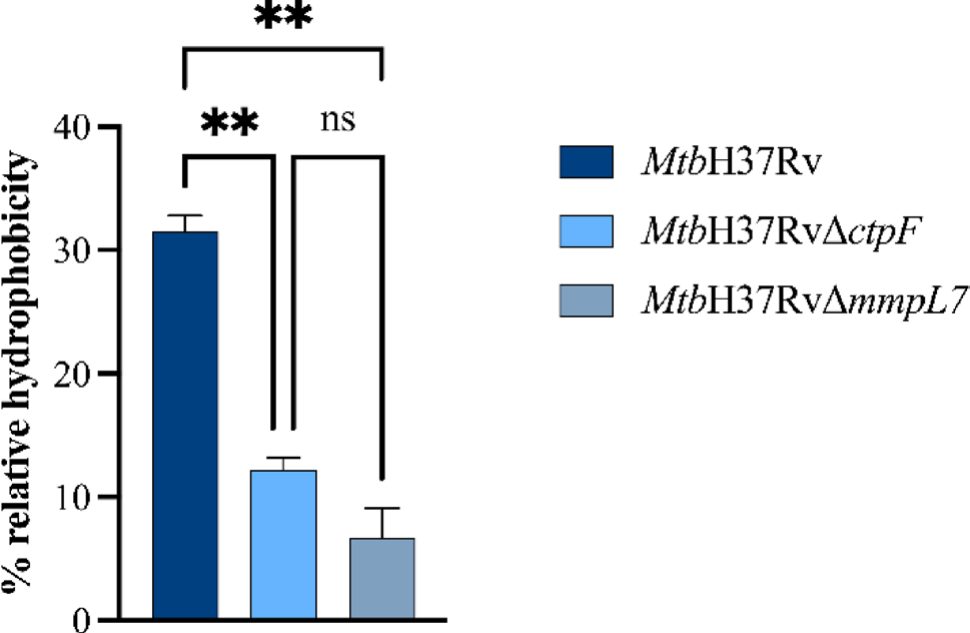
Percentage of relative hydrophobicity of mycobacterial cells after hexadecane partitioning. The plotted values represent the means ± standard error of the mean (SEM) from three independent experiments for each strain. Asterisks indicate statistical significance based on one-way ANOVA followed by Tukey’s post hoc test (ns *p* > 0.05; **p < 0.01). Statistical analyses were performed using GraphPad Prism version 9.0.0 for Windows (GraphPad Software, San Diego, CA, USA)

Given the well-established relationship between surface lipid composition, cell surface hydrophobicity, and virulence in *Mtb* strains (Jankute et al. 2017), we examined the effects of *ctpF* and *mmpL7* gene deletions on *Mtb* hydrophobicity. To this end, a partitioning assay was performed in which suspended mycobacterial cells were distributed between an aqueous phase and an organic phase. The mutant strains exhibited a lower percentage of relative hydrophobicity compared to the parental strain (Fig. 3), suggesting that mutations alter the cell wall lipid composition.

### A mutation in the *ctpF* gene impairs the *Mtb *capacity for biofilm formation and colony spreading

**Fig. 4:**
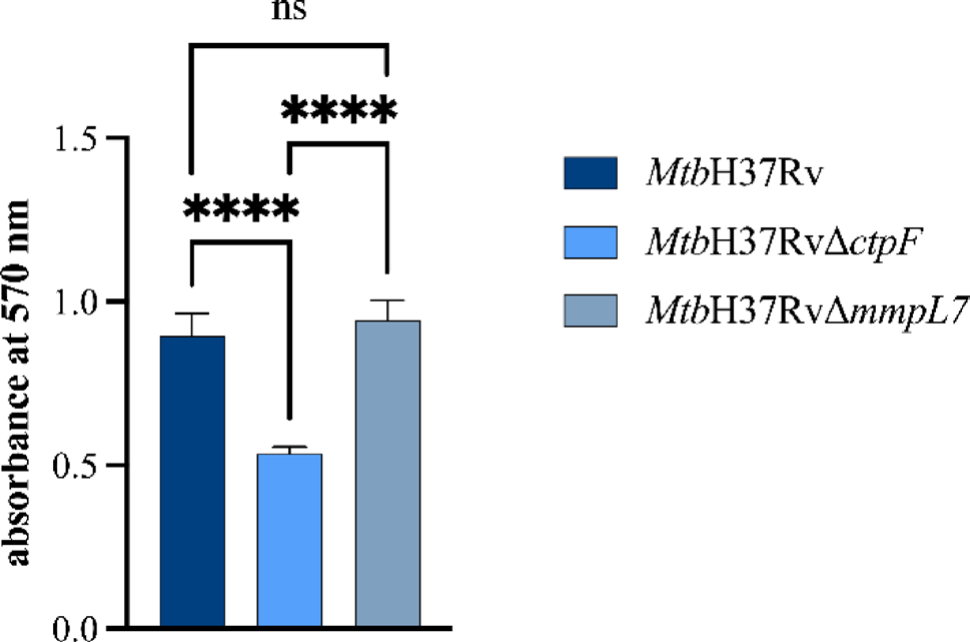
Quantification of biofilm formation in *Mtb* strains at the liquid–air interface by crystal violet staining and measured at 570 nm. Plotted values represent the means ± SEM from four independent experiments. Asterisks indicate statistical significance based on one-way ANOVA followed by Tukey’s post hoc test (ns p>0.05;****p<0.0001). Statistical analyses were performed using GraphPad Prism version 9.0.0 for Windows (GraphPad Software, San Diego, CA, USA)

Colony spreading and biofilm formation have been associated with colonial morphology and the composition of cell envelope lipids in *Mycobacterium* strains. Consequently, we observed that the *Mtb*H37Rv∆*ctpF* strain exhibited a substantially diminished capacity for biofilm formation at the liquid-air interface in comparison with *Mtb*H37Rv (****p < 0.0001) and *Mtb*H37Rv∆*mmpL7* strains (****p < 0.0001). In contrast, there was no significant difference in biofilm formation capacity between *Mtb*H37RvΔ*mmpL7* and *Mtb*H37Rv (*p* = 0.4880) (Fig. 4).

**Fig. 5:**
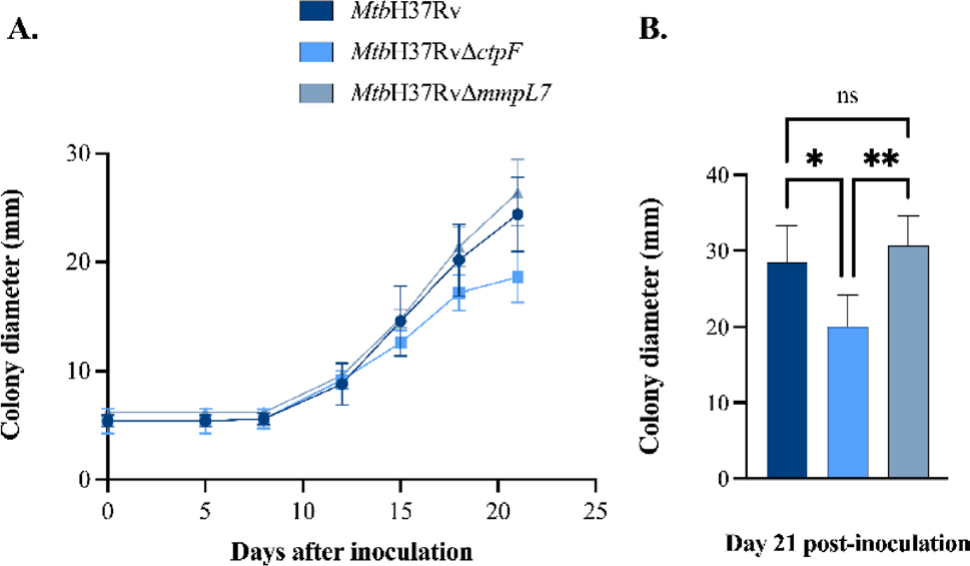
Colony spreading of *Mtb* strains on 7H9-OADC medium solidified with 0.4% agarose. **A.** Colony spreading was monitored by measuring the diameter of radial growth for 4 weeks. **B.** The plotted values represent the means ± SEM from five independent experiments per strain at 21 days post-inoculation. Asterisks indicate statistical significance based on one-way ANOVA followed by Tukey’s post hoc test (ns *p* > 0.05; *p < 0.05; **p < 0.01). Statistical analyses were performed using GraphPad Prism version 9.0.0 for Windows (GraphPad Software, San Diego, CA, USA)

In addition, colony spreading significantly changed at the end of the second week post-inoculation in *Mtb* strains (Fig. 5A). From this point onward, a noticeable increase in the colony diameter was observed, resulting in differences in the size of the spreading halo among the different strains. Consistent with these observations, the *Mtb*H37RvΔ*ctpF* strain exhibited reduced colony spreading compared to the *Mtb*H37Rv (*p = 0.0268) and *Mtb*H37RvΔ*mmpL7* (**p = 0.0065) strains (Fig. 5B), suggesting a stronger interaction between the cell surface components of this strain and the culture medium.

### Deletion of *ctpF* and *mmpL7* alters the lipid profile of *Mtb*H37Rv

**Fig. 6:**
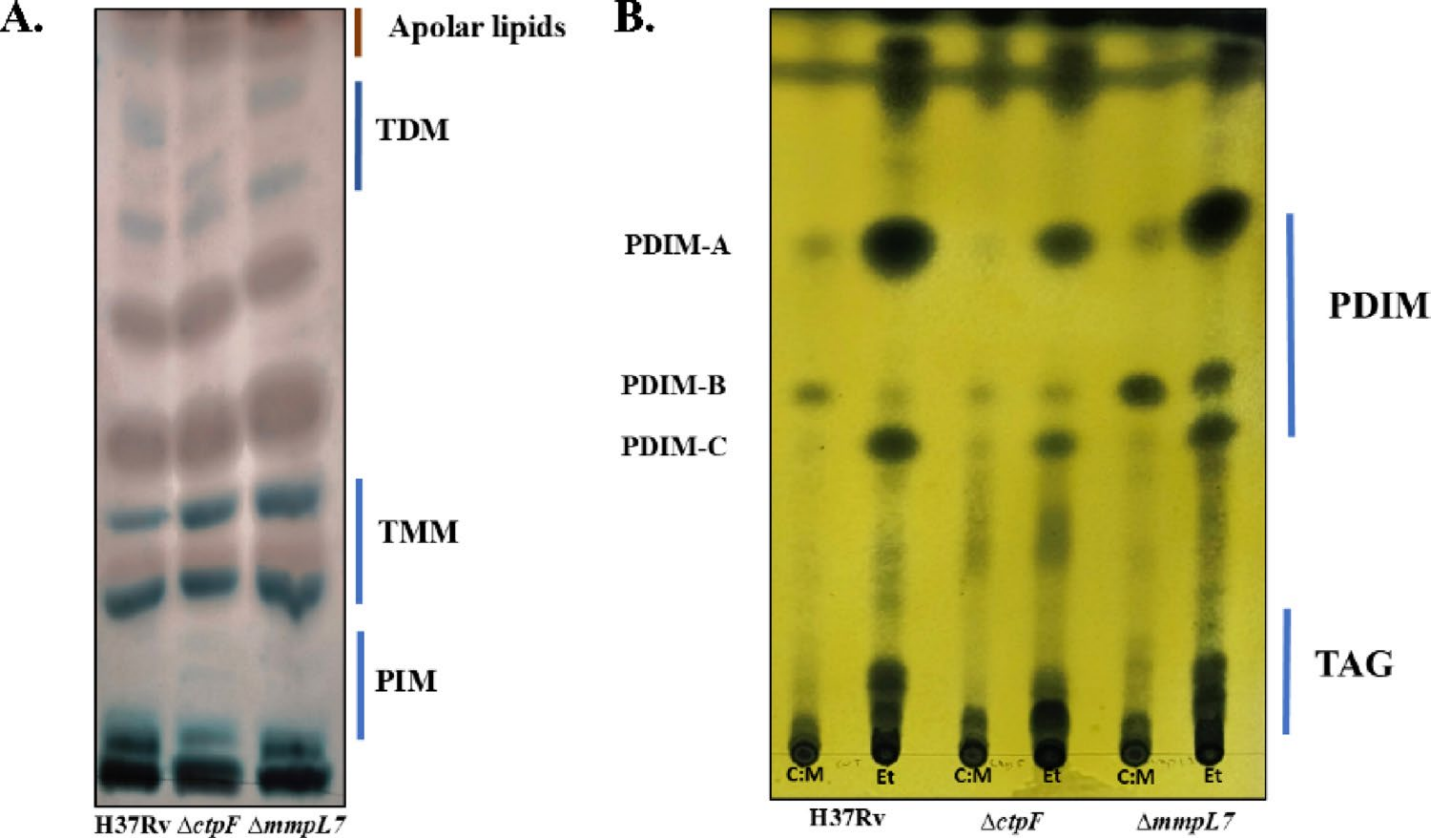
TLC of lipid extracts obtained from *Mtb*H37Rv, *Mtb*H37Rv∆*ctpF*, and *Mtb*H37Rv∆*mmpL7* strains. **A.** Glycolipids were extracted using chloroform–methanol. TLC was developed using (65:25:4) chloroform: methanol: water as the mobile phase and revealed with 1% anthrone in concentrated sulfuric acid. **B.** Lipids were extracted with chloroform-methanol (C:M) or petroleum ether (Et). TLC was developed with (9:1) Et: diethyl ether as the mobile phase and revealed using 10% phosphomolybdic acid

Analysis of the lipid components of the *Mtb* cell envelope is particularly relevant for understanding microbiological traits associated with virulence in mutant strains. Accordingly, a preliminary characterization of the mycobacterial lipid profile was performed. The lipids were identified based on their reported mobility on silica plates (Sacco et al. 2010; Pandey et al. 2023). As shown in Fig. 6A, glycolipids such as phosphatidylinositol mannosides (PIM) and trehalose 6,6′-dimycolate (TDM), or “cord factor” were detected.

Specifically, TDM profiles of the *Mtb*H37RvΔ*ctpF* strain differed from those of *Mtb*H37Rv and the amount of highly polar PIMs (near the inoculation point) decreased in *Mtb*H37RvΔ*ctpF* (Fig. 6A). Also, *Mtb*H37RvΔ*ctpF* showed a shift in one of the TDM bands, where the less polar signal exhibited reduced mobility relative to the *Mtb*H37Rv and *Mtb*H37RvΔ*mmpL7* strains. In contrast, the *Mtb*H37RvΔ*mmpL7* strain displayed a PIM and TDM profile similar to *Mtb*H37Rv, with bands of comparable intensity and retention factor. No changes were detected in the trehalose monomycolate (TMM) region among the *Mtb* strains (Fig. 6A).

Conversely, TLC of triacylglycerol (TAG) and PDIM of *Mtb*H37Rv, *Mtb*H37RvΔ*ctpF*, and *Mtb*H37RvΔ*mmpL7* showed similar qualitative profiles (Fig. 6B). Three distinct PDIM bands were observed, likely corresponding to structural variants differing in phthiocerol chain length and in the presence of either an ester or a ketone group (Camacho et al. 2001). PDIM exists in multiple structural forms, including PDIM-A, PDIM-B, and PDIM-C, the latter of which remains chemically uncharacterized (Siméone et al. 2007; Chauhan et al. 2013; Krishnamoorthy et al. 2021; Lu et al. 2024).

Although the overall PDIM pattern appeared similar among strains, the *Mtb*H37RvΔ*mmpL7* extract displayed a more intense PDIM-B band in both extraction systems (chloroform–methanol and petroleum ether) (Fig. 6B). This observation suggests a qualitative shift in the relative distribution of PDIM species; however, no densitometric or mass spectrometry quantification was performed in this study.

### Genes involved in PDIM biosynthesis are downregulated in the *Mtb*H37Rv∆*ctpF* and *Mtb*H37Rv∆*mmpL7* strains

**Fig. 7:**
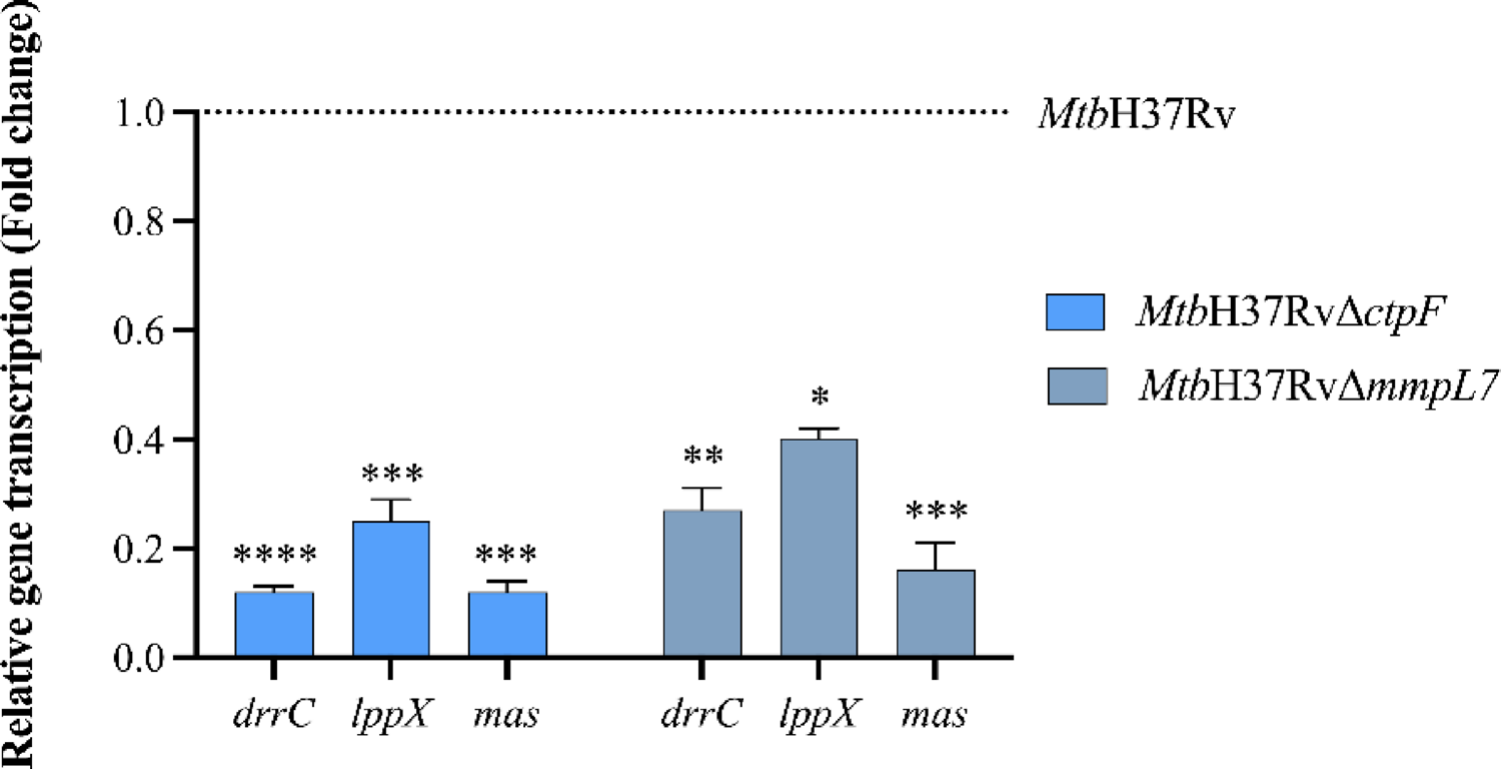
Transcriptional profile of some genes involved in the biosynthesis and transport of PDIM in *Mtb* strains. Transcription levels are expressed as the ratio of gene expression relative to the mutant strain (*Mtb*H37RvΔ*mmpL7* and *Mtb*H37RvΔ*ctpF*) to the control strain (*Mtb*H37Rv, transcription ratio ≈ 1.0). Relative gene transcription was normalized to *16 S rRNA *(housekeeping gene). The plotted values represent the mean ± standard deviation from four technical replicates. Asterisks indicate statistical significance determined by two-way ANOVA followed by Sidak’s multiple comparisons test (ns *p* > 0.05; *p < 0.05; **p < 0.01; ***p < 0.001; ****p < 0.0001). Statistical analyses were performed using GraphPad Prism version 9.0.0 for Windows (GraphPad Software, San Diego, CA, USA)

To assess whether deletion of *ctpF* and *mmpL7* affects the transcription of genes involved in biosynthesis and transport of PDIM, an established virulence determinant (Cox et al. 1999; Jain and Cox 2005; Giovannini et al. 2012; Quigley et al. 2017; Rens et al. 2021), the expression levels of *mas*, *drrC*, and *lppX* were quantified by qRT-PCR. As shown in Fig. 7, all three genes were significantly downregulated in both mutant strains compared with the parental strain. Notably, transcriptional repression was more pronounced in *Mtb*H37RvΔ*ctpF*, suggesting that disruption of *ctpF* (which encodes a Ca^2+^-ATPase), exerts a broader regulatory influence on PDIM-associated genes. Furthermore, the reduced expression of *mas*, *drrC*, and *lppX* in *Mtb*H37RvΔ*mmpL7* indicates that disruption of *mmpL7*, a transporter implicated in PDIM export, also affects the transcription of genes involved in PDIM-related functions.

### *Mtb*H37Rv*∆ctpF* and *Mtb*H37Rv*∆mmpL7* maintain the ability to secrete the immunomodulatory antigens ESAT-6 and CFP-10

**Fig. 8:**
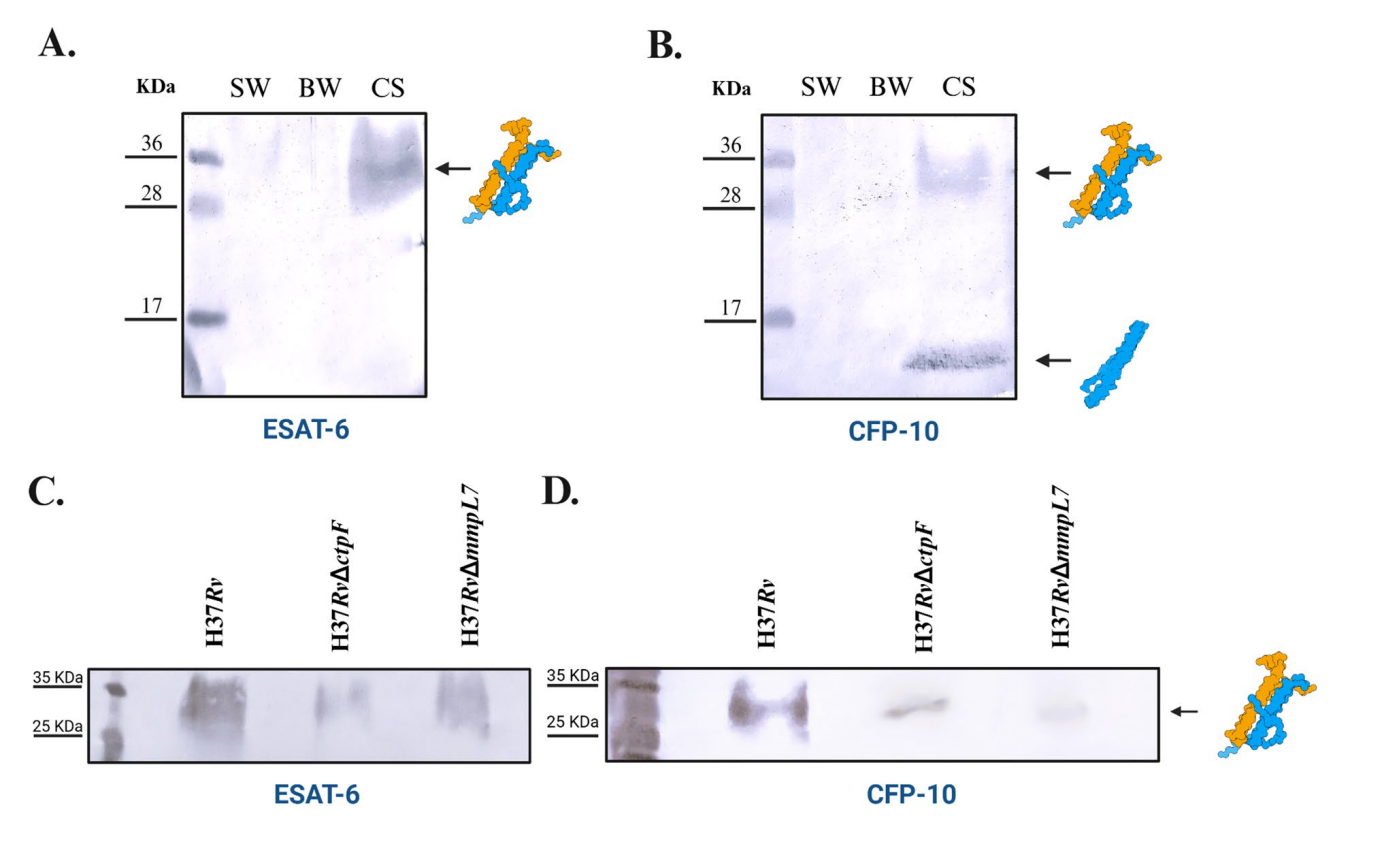
Immunodetection of ESAT-6 and CFP-10 antigens in *Mtb* strains (15 µg protein). **A.** ESAT-6 and **B.** CFP-10 extracted from *Mtb*H37Rv whole-cell lysate by sonication (SW) or bead beating disruption (SB), and culture supernatant (CS). **C.** ESAT-6 and **D.** CFP-10 obtained from the culture supernatant of *Mtb*H37Rv, *Mtb*H37RvΔ*ctpF*, and *Mtb*H37RvΔ*mmpL7* strains . Detection was performed using primary antibodies anti-*Mtb* ESAT-6 and anti-*Mtb* CFP-10 at 0.5 µg/mL, followed by the secondary antibody anti-rabbit IgG (whole molecule)–alkaline phosphatase conjugate at a dilution of 1:20000. Signal development was achieved using the NBT-BCIP solution. Molecular weight marker: prestained Protein Ladder (250 kDa). Diagram created in https://BioRender.com

Given the critical roles of ESAT-6 and CFP-10 during *Mtb* infection (Anes et al. 2023) and their reported association with the PDIM (Augenstreich et al. 2017), we evaluated whether the deletion of *ctpF* or *mmpL7* affects the production of these antigens in the mutant strains. In *Mtb*H37Rv strain, ESAT-6 and CFP-10 antigens were not detected in whole-cell extracts obtained by sonication or bead beating. This is likely due to their low intracellular abundance and the limited sensitivity of the colorimetric detection method employed (Fig. 8A). Therefore, the analyses focused on the secreted fraction in the culture supernatant detected between 28 and 36 kDa as an ESAT–6–positive band (Fig. 8A). CFP-10 showed two bands: one in the same range (28 and 36 kDa) and another at approximately 10 kDa (Fig. 8B). Moreover, *Mtb*H37RvΔ*ctpF* and *Mtb*H37RvΔ*mmpL7* strains also secreted ESAT-6 with bands detected between 28 and 36 kDa (Fig. 8C). Similarly, CFP-10 was identified in mutant strains showing two bands, one between 28 and 36 kDa and another near 10 kDa (Fig. 8D).

## Discussion

This study integrated phenotypic and molecular features in *Mtb* strains defective in the plasma membrane transporters CtpF and MmpL7. The mutant strains exhibited a marked reduction in colonial rugosity compared with the parental strain (Fig. 2). Colony roughness in *Mtb* has been associated with the presence of elongated, serpentine surface structures known as the “cord factor” and is a characteristic feature of virulent *Mtb* strains (Lerner et al. 2020; Gong et al. 2023). Previous studies have shown that mutant *Mtb* strains with altered PDIM levels form colonies with a smoother morphology and display attenuated virulence compared with the *Mtb*H37Rv strain (Cox et al. 1999; Giovannini et al. 2012). Accordingly, the reduced colony rugosity observed in the *Mtb*H37RvΔ*ctpF* and *Mtb*H37RvΔ*mmpL7* strains is consistent with alterations in cell envelope lipid composition, which in turn influence overall cell surface hydrophobicity and virulence-associated phenotypes (Hunter et al. 2006). Consistently, both mutant strains exhibited a marked reduction in cell surface hydrophobicity compared to the *Mtb*H37Rv strain (Fig. 3). This likely reflects either a diminished level or an altered spatial distribution of surface lipids in the mycobacterial envelope. In this context, a colony spreading assay was employed to evaluate the impact of bacterial surface properties on the ability of cells to disperse across 7H9–OADCagarose plates (Fig. 5). The *Mtb*H37RvΔ*ctpF* strain exhibited a markedly reduced capacity to spread over the agarose surface, a phenotype that can be attributed to its decreased cell surface hydrophobicity compared with the parental strain. In contrast, the *Mtb*H37RvΔ*mmpL7* strain displayed a spreading capacity comparable to that of *Mtb*H37Rv, despite its reduced cell surface hydrophobicity.

A similar trend was observed in the biofilm formation assay, in which the *Mtb*H37RvΔ*ctpF* strain exhibited a significantly reduced biofilm-forming capacity compared with *Mtb*H37Rv and *Mtb*H37RvΔ*mmpL7* strains, which did not differ significantly from each other (Fig. 4). To further elucidate the phenotypes observed in the mutant strains, we analyzed cell wall lipid profiles associated with these cellular processes. As shown in Fig. 6A, the *Mtb*H37RvΔ*ctpF* strain exhibited alterations in the TDM profile. These changes may result from the synthesis of TDM species containing mycolic acid chains with altered conformations, leading to differences in polarity (Fig. 6A). Such alterations are consistent with the observed changes in colony roughness, as a reduced abundance of cord factor that suggests decreased incorporation and/or structural modification of TDM within the cell envelope of *Mtb*H37RvΔ*ctpF*. These effects may be linked to perturbations in intracellular Ca^2+^ homeostasis, which could influence multiple cellular processes and regulatory pathways (Kruh et al. 2008; Campbell 2015; Yeruva et al. 2016).

Conversely, the *Mtb*H37RvΔ*mmpL7* strain exhibited an apparent shift in PDIM-B band intensity (Fig. 6B), consistent with the role of MmpL7 in PDIM export to the cell surface. Accordingly, disruption of *mmpL7* results in intracellular retention of PDIMs as previously reported (Cox et al. 1999; Camacho et al. 2001). Impaired PDIM translocation is consistent with a contribution to the smoother colony morphology observed in *Mtb*H37RvΔ*mmpL7* and may also underlie its attenuated virulence (Cox et al. 1999; Giovannini et al. 2012).

Importantly, impaired PDIM transport resulting from *mmpL7* deletion and altered Ca^2+^ homeostasis associated with *ctpF* deletion may converge to alter membrane organization and host–pathogen interactions. While PDIM retention can alter lipid distribution in the cell envelope (Jain and Cox 2005; Sulzenbacher et al. 2006), the disruption of Ca^2+^ balance may affect enzymatic activities and stress signalling pathways (Domínguez 2018). The perturbation of lipid architecture and ion homeostasis could therefore compromise coordinated responses required for intracellular persistence.

A biologically plausible connection between altered Ca^2+^ homeostasis and lipid biosynthesis involves changes in precursor availability. Polyketide synthases, including those responsible for PDIM synthesis (PpsA–E), require malonyl-CoA (Azad et al. 1997) and other acyl-CoA derivatives, which are produced via acetyl-CoA carboxylase (ACC) (Polyak et al. 2012; Tong 2013). The carboxylation reaction requires ATP and Mg^2+^ to stabilize the negative charges of phosphates and facilitate the enzymatic mechanism (Polyak et al. 2012). Depending on their intracellular concentrations, Ca^2+^ and Mg^2+^ compete for protein-binding sites (Dudev and Lim 2014). The alteration of Ca^2+^ homeostasis may influence Mg^2+^-dependent metabolic processes, potentially affecting precursor availability for lipid biosynthesis, reducing malonyl-CoA availability, and modifying the lipid profile.

Although genetic complementation represents an important approach to confirm the relationship between gene deletion and phenotype, complementation of the *ctpF* and *mmpL7* mutants proved technically challenging. Complementation was initially attempted using the vector pMV261 under the strong *hsp60* promoter; however, the resulting strains displayed abnormal growth behavior and failed to restore WT phenotypes (Maya-Hoyos 2021). Because physiological expression levels are critical for membrane transporters, further complementation strategies using integrative vectors under moderate promoters are currently under development. Nevertheless, the phenotypes described here are consistent with the established roles of MmpL7 in PDIM export (Cox et al. 1999; Camacho et al. 2001) and of CtpF, a Ca^2+^ P-type ATPase (Maya-Hoyos et al. 2019). The reproducibility of independent assays supports the validity of the comparison between mutant and WT strains.

To complement the lipid analyses, transcriptional profiling was performed for selected genes *(lppX*, *drrC*, and *mas*) involved in PDIM biosynthesis and transport (Astarie-Dequeker et al. 2009; Cambier et al. 2014). The *drrC* gene encodes a component of an ATP-binding cassette (ABC) transporter complex that functions in concert with MmpL7 to mediate PDIM export to the cell surface (Moolla 2020). Consistent with this role, *Mtb* strains lacking *mmpL7* can synthesize PDIM in the cytosol but fail to translocate it to the periplasm, typically exhibiting reduced expression of PDIM transport-associated genes, as observed in this study (Fig. 7) (Camacho et al. 1999; Cox et al. 1999).

Similarly, *lppX*, which encodes a periplasmic lipoprotein required for PDIM localization at the cell surface through direct protein-lipid interactions (Sulzenbacher et al. 2006), displayed reduced expression in both mutant strains. In addition, *mas*, encoding the polyketide synthase responsible for generating mycocerosic acid precursors esterified to the phthiocerol backbone of PDIM (Jain and Cox 2005), also exhibited decreased transcriptional levels in both mutants (Fig. 7). The transcriptional analysis revealed reduced expression of these genes *(lppX*, *drrC*, and *mas*) in the *Mtb*H37RvΔ*ctpF* and *Mtb*H37RvΔ*mmpL7* strains compared to the WT strain. These changes are consistent with the altered lipid profiles observed in the mutant strains and reflect a coordinated cellular response to perturbations in envelope composition. However, these transcriptional changes should be interpreted as associative responses rather than direct evidence of regulatory mechanisms. Although this transcriptional repression may reflect feedback responses to lipid disequilibrium or cell envelope remodeling, the precise regulatory mechanisms linking transporter disruption to these transcriptional changes remain unclear. Another interesting aspect is to consider whether mutations in *ctpF* and *mmpL7* affect the production of immunodominant antigens such as ESAT-6 and CFP-10. It is well established that ESAT-6 and CFP-10 form a heterodimeric protein complex (Renshaw et al. 2002), which functions to modulate phagosome maturation (Tan et al. 2006). The immunoreactive band detected between 28 and 36 kDa in-culture supernatant represents a higher-order complex (Fig. 8). Given that ESAT-6 and CFP-10 are known to form a tight 1:1 heterodimer (Renshaw et al. 2002), this signal may correspond to a preserved or reassociated ESAT-6/CFP-10 complex. Notably, ESAT-6 has been reported to form self-associated complexes and to exhibit structural stability under certain conditions (Daugelat et al. 2003; Bates et al. 2024), which may explain the altered migration pattern observed (Fig. 8). However, because the analyses were performed under denaturing SDS-PAGE conditions, the precise composition of the detected band cannot be conclusively determined in the present study. Native PAGE, chemical crosslinking, or mass spectrometry will be required to confirm its identity.

Considering the objectives of the present study, and given that ESAT-6 and CFP-10 are major *Mtb* antigens associated with virulence and robust T-cell activation (Brandt et al. 1996; Brodin et al. 2005; Wu et al. 2008), the evaluation of their production in the mutant strains is of particular relevance, as it further supports their relevance in the context of potential vaccine strategies, despite incomplete characterization of their conformational states. The relationship between PDIM and ESX-1 has been recognized as a coordinated virulence mechanism rather than independent pathways. Previous studies have demonstrated that PDIM-deficient strains exhibit altered ESX-1–mediated secretion and reduced phagosomal disruption, supporting a functional interplay between lipid composition and protein secretion systems (Jones et al. 2024; Weaver et al. 2026). In this context, the preservation of ESAT-6 and CFP-10 secretion observed in our mutants suggests that ESX-1 activity remains functional despite alterations in PDIM-associated pathways.

Although the present study does not include macrophage or animal infection experiments, several of the phenotypes observed including altered lipid profiles and changes in virulence-associated gene expression, are widely recognized as surrogate markers of pathogenic potential in *Mtb.* Therefore, these findings provide mechanistic insights that complement previous infection-based studies of individual mutants and justify future construction of a double mutant strain.

## Conclusions

The findings of this work indicate that *Mtb*H37RvΔ*ctpF* and *Mtb*H37RvΔ*mmpL7* mutants exhibit phenotypic traits consistent with altered virulence-associated characteristics. Both mutants displayed modifications in colony morphology and surface hydrophobicity, reflecting structural remodeling of the cell envelope. In agreement with these observations, transcriptional profiling revealed reduced expression of genes involved in PDIM metabolism, in line with the altered lipid profiles detected. However, the regulatory mechanisms underlying this association remain to be elucidated. Notably, both mutants preserved the secretion of the antigens ESAT-6 and CFP-10, suggesting that key immunogenic features are maintained despite these envelope-associated alterations.

This study has some limitations that should be considered. The analyses were restricted to in vitro characterization and do not directly reflect bacterial behavior in infection models. Furthermore, lipid profiling was based on qualitative TLC, and ESX-1 activity was assessed indirectly through ESAT-6 and CFP-10 detection. In addition, restoration of WT phenotypes under physiological conditions was not achieved through functional complementation. Taken together, these limitations indicate that the observed phenotypes should be interpreted as indicative of altered microbiological traits rather than direct evidence of virulence attenuation. Nevertheless, the results support an association between membrane transport processes and traits commonly linked to virulence in *Mtb.*

## Electronic Supplementary Material

Below is the link to the electronic supplementary material.

Supplementary Material 1

## Data Availability

No datasets were generated or analysed during the current study.

## Abbreviations

AES: Allelic exchange substrate
Amp: Ampicillin
BCG: Bacillus Calmette–Guérin
BCIP: 5-bromo-4-chloro-3-indolyl-phosphate
CFP-10: Culture filtrate protein-10
CtpF: Ca^2+^ P-type ATPase
DEPC: Diethyl pyrocarbonate
DTT: Dithiothreitol
ESAT-6: Early secreted antigenic target-6
ESX-1: ESAT-6 secretion system 1
HIV: Human immunodeficiency virus
Hyg: Hygromycin
IPTG: Isopropyl β-D-1-thiogalactopyranoside
Kan: Kanamycin
LB: Luria–Bertani medium
MDR: Multidrug-resistant
MmpL: Mycobacterial membrane protein Large
MmpL7: Mycobacterial membrane protein Large 7
*Mtb*: *Mycobacterium tuberculosis*
*Mtb*H37Rv: Virulent reference strain of *Mtb*
*Mtb*H37RvΔ*ctpF*: *ctpF* deletion mutant of *Mtb*H37rv
*Mtb*H37RvΔ*mmpL7*: *mmpL7* deletion mutant of *Mtb*H37rv
NBT: Nitro blue tetrazolium
OADC: Oleic acid–albumin–dextrose–catalase
OD_600_: Optical Density at 600 nm
PBS: Phosphate-buffered saline
PDIM: Phthiocerol dimycocerosate
PIM: Phosphatidylinositol mannoside
SEM: Standard error of the mean
TAG: Triacylglycerol
TB: Tuberculosis
TDM: Trehalose 6,6′-dimycolate
TLC: Thin-layer chromatography
TMM: Trehalose monomycolate
Tyl: Tyloxapol
WHO: World Health Organization
WM: Molecular weight marker
WT: Wild-type
XDR: Extensively drug-resistant
X-Gal: 5-bromo-4-chloro-3-indolyl β-D-galactopyranoside
