# Supplementary material for: Mycobacterium tuberculosis lacking CtpF and MmpL7 transporters exhibits altered microbiological traits associated with virulence determinants
Source: Arch Microbiol. 2026 May 9;208(8):381. doi: 10.1007/s00203-026-04921-7 (PMC13157411; doi:10.1007/s00203-026-04921-7)
Supplement: Supplementary file 1 — Supplementary Material 1 [file 203_2026_4921_MOESM1_ESM.docx]

**Table S1. Bacterial strains, plasmids, and primers used in this study.**

| **Strains** | | **Relevant features** | **Reference** |
| --- | --- | --- | --- |
| ***E.coli*** | | | |
| JM109 | *recA-, endA-,* Blue/white color screening with *lac*Z∆M15 | | Thermo Fisher Scientific |
| HB101 | *F–, thi-1, hsdS20 (r_B_^–^, m_B_^–^), supE44, recA13, ara-14, leuB6, proA2, lacY1, galK2, rpsL20 (str^r^), xyl-5, mtl-1.* | | Promega |
| ***Mtb*** | | | |
| *Mtb*H37Ra | Slow-growing attenuated wild type strain, Amp^R^, Chx | | ATCC25177 |
| *Mtb*H37Rv | *Mtb* virulence reference, Amp^R^, Chx^R^ | | ATCC27294 |
| *Mtb*H37Rv::pJV53 | Mutant of *ctpF* gene (replaced by Hyg^R^ cassette) with the pJV53 plasmid (recombineering strain) | | [(Maya-Hoyos et al. 2022)](https://www.zotero.org/google-docs/?MXiAEd) |
| *Mtb*H37RvΔ*ctpF* | Mutant of *ctpF* gene (replaced by Hyg^R^ cassette) | | [(Maya-Hoyos et al. 2022)](https://www.zotero.org/google-docs/?Snu6NO) |
| *Mtb*H37RvΔ*mmpL7* | Mutant of *mmpL7* gene (replaced by Hyg^R^ cassette) | | This study |
| **Plasmids** | | **Relevant features** | **Reference** |
| pGEM ®-T Easy | Cloning vector for PCR products, Blue/white color screening | | Promega |
| pYUB854 | The HygR cassette is flanked by the γδ-res sites and by two MCSs for directional cloning | | [(Bardarov et al. 2002)](https://www.zotero.org/google-docs/?tDuyvZ);  Gift from Universidad de Zaragoza |
| pVVG4 | 520 pb upstream and 560 pb downstream of *MtbRv2942* (*mmpL7*) in pYUB854, Hyg^R^ | | This study |
| **Primer** | **Sequence (5’-3’)** | | **Reference** |
| MmpL7 int dir | GGCTAGGTGTCTTTGTGGCA | | This study |
| MmpL7 int rev | GAGATTCTTCAGCATCGCCG | | This study |
| Comp Up Dir | GTTCTTCGTGCCGCTAGTTC | | This study |
| pYUB Rev | GTGGCTCCCTCACTTTCTGG | | [(Maya-Hoyos et al. 2019)](https://www.zotero.org/google-docs/?rL2Vwy) |
| HygDirOut | GAACTAGCGGCACGAAGAAC | | [(Maya-Hoyos et al. 2019)](https://www.zotero.org/google-docs/?XDAwCI) |
| CompDowmRev | GATAGGCCTGCAACAACTCC | | This study |
| 16SrRNAd | GAGATAGGCGTTCCCTTGTG | | [(Maya-Hoyos et al. 2015)](https://www.zotero.org/google-docs/?YgQWfx) |
| 16SrRNAr | CTGGACATAAGGGGCATGAT | | [(Maya-Hoyos et al. 2015)](https://www.zotero.org/google-docs/?VJluAV) |
| RT-mmpL7 Dir | CTGGCCGTTATCTTGCTCAC | | This study |
| RT-mmpL7 Rev | CGATACGACCGGCTTTGATG | | This study |
| RT-lppX Dir | GACCACCAAAATCACCGGGA | | This study |
| RT-lppX Rev | GAGCTGAATCGACCCGGATC | | This study |
| RT-mas Dir | ACCATGGAGCAAACCTACGG | | This study |
| RT-mas Rev | GAATTCACTTGCTTGGCGGG | | This study |
| RT-drrC Dir | GTTTCGACAAGGCCTGATCC | | This study |
| RT-drrC Rev | ACCCGTGGAGAAGAAGATCG | | This study |
